# Role of boron and its interaction with other elements in plants

**DOI:** 10.3389/fpls.2024.1332459

**Published:** 2024-02-12

**Authors:** Peter Vera-Maldonado, Felipe Aquea, Marjorie Reyes-Díaz, Paz Cárcamo-Fincheira, Braulio Soto-Cerda, Adriano Nunes-Nesi, Claudio Inostroza-Blancheteau

**Affiliations:** ^1^Programa de Doctorado en Ciencias Agropecuarias, Facultad de Recursos Naturales, Universidad Católica de Temuco, Temuco, Chile; ^2^Laboratorio de Bioingeniería, Facultad de Ingeniería y Ciencias, Universidad Adolfo Ibáñez, Santiago, Chile; ^3^Departamento de Ciencias Químicas y Recursos Naturales, Facultad de Ingeniería y Ciencias, Universidad de La Frontera, Temuco, Chile; ^4^Center of Plant, Soil Interaction and Natural Resources Biotechnology, Scientific and Technological Bioresource Nucleus (BIOREN), Universidad de La Frontera, Temuco, Chile; ^5^Laboratorio de Fisiología y Biotecnología Vegetal, Departamento de Ciencias Agropecuarias y Acuícolas, Facultad de Recursos Naturales, Universidad Católica de Temuco, Temuco, Chile; ^6^Nucleo de Investigación en Producción Alimentaria, Facultad de Recursos Naturales, Universidad Católica de Temuco, Temuco, Chile; ^7^Departamento de Biologia Vegetal, Universidade Federal de Viçosa, Viçosa, MG, Brazil

**Keywords:** boron, interaction, mineral elements, low pH, protein transport, oxidative stress

## Abstract

Boron (B) is an essential microelement for plants, and its deficiency can lead to impaired development and function. Around 50% of arable land in the world is acidic, and low pH in the soil solution decreases availability of several essential mineral elements, including B, magnesium (Mg), calcium (Ca), and potassium (K). Plants take up soil B in the form of boric acid (H_3_BO_3_) in acidic soil or tetrahydroxy borate [B(OH)_4_]^-^ at neutral or alkaline pH. Boron can participate directly or indirectly in plant metabolism, including in the synthesis of the cell wall and plasma membrane, in carbohydrate and protein metabolism, and in the formation of ribonucleic acid (RNA). In addition, B interacts with other nutrients such as Ca, nitrogen (N), phosphorus (P), K, and zinc (Zn). In this review, we discuss the mechanisms of B uptake, translocation, and accumulation and its interactions with other elements, and how it contributes to the adaptation of plants to different environmental conditions. We also discuss potential B-mediated networks at the physiological and molecular levels involved in plant growth and development.

## Introduction

Boron (B) is an essential element for growth, development, productivity and quality of crops (Wang et al., 2015; Shireen et al., 2018; Pereira et al., 2021). It is found in soils as boric acid [B(OH)_3_] and tetrahydroxy borate [B(OH)_4_]^-^, and is distributed unevenly in soil solution and in organic and mineral fractions depending on the soil pH (Hrmova et al., 2020). Boron is considered as the most mobile, and often one of the most deficient, microelements in soils (Wimmer and Eichert, 2013; Hrmova et al., 2020). Plant absorbs B as [B(OH)_3_] via the channels in the plasma membrane, and export [B(OH)_4_]^-^ through specific transporters (Stangoulis et al., 2001; Yoshinari and Takano, 2017). The availability of B in soils depends on adsorption-desorption processes, which are influenced by various physicochemical characteristics such as soil pH, texture, moisture, clay content and type of clay minerals, hydroxy-oxides of aluminum (Al) and iron (Fe), calcium carbonate (CaCO_3_), and organic matter (Arora and Chahal, 2010). A positive correlation has been reported between B adsorption on clay minerals or Al hydroxy-oxides and soil pH (Keren, 1996). At pH below 7.0, the dominant B form [B(OH)_3_] shows relatively low affinity for clay, but in the alkaline pH range, the proportion of borate increases rapidly, reaching maximum adsorption around pH 9.0 (Elrashidi and O’Connor, 1982).

Boron is a microelement and its concentration in dried leaf tissue varies depending on species and genotypes (Arunkumar et al., 2018). Boron participates in cell wall biosynthesis and structural integrity (Shireen et al., 2018; Pereira et al., 2021), mainly related to the formation of borate esters with rhamnogalacturonan (RG‐II) that improve the porosity and elasticity of the cell wall (Funakawa and Miwa, 2015; Nejad and Etesami, 2020). In *Arabidopsis thaliana* roots, B is essential for the crosslinking of cell wall RG-II and pectin assembly (Camacho-Cristóbal et al., 2008). In addition, it is also involved in the stimulation of reproductive tissues, improvement of seed quality, ion traffic through the membranes, cell division and elongation, protein cytoskeletal function, the metabolism of antioxidants, ascorbic acid and polyphenols, sugar transport, oxidoreductase activity, and the biosynthesis and transport of plant hormones, among other processes (Lu et al., 2015; Shireen et al., 2018).

By comparing the B concentrations in plants, it has been observed, for example, that an optimal B concentration enhances H^+^-ATPase activity, thus maintaining the electrochemical gradients across the plasma membrane; by contrast, under limited availability of B, reduced H^+^-ATPase activity is found in plasma membrane-enriched vesicles isolated from *Cicer arietinum* roots (Shireen et al., 2018). Under B deficiency, an excessive accumulation of soluble sugars has been observed in the plant leaves by a reduction in the photosynthates translocation (Camacho-Cristóbal et al., 2004). This could affect increasing the concentration of phenolic compounds and others derivatives, like quinones, which may be oxidized and exacerbate reactive oxygen species (ROS) production, including oxygen radicals (Han et al., 2008).

Two functionallly different kinds of transporters have been identified in plant cells: boron transporters (BORs) that have a B export function in plant cells, and nodulin-26-like intrinsic protein (NIPs) members of the major intrinsic proteins (MIP) family, that include some boric acid channels (Wang et al., 2015; Zhang et al. 2022; Pereira et al., 2021). *BOR1* was first reported in *A. thaliana* and is necessary for effective transport in the xylem, preferentially for the translocation of B into younger parts of plants (Takano et al., 2001; Takano et al., 2002). Additionally, aquaporins in the NIP subgroup have been identified as boric acid channels required for plant growth under B deficiency. The *NIP5;1* transporter gene is expressed in plasma membrane of root epidermis cortical and endodermal cells for boric acid transported, whereas the boric acid channel NIP6;1 is involved in the B transport for proliferative plant tissues (Zhou et al., 2015).

Boron interacts with other mineral elements, influencing several physiological and biochemical processes (Tariq and Mott, 2007). In particular, B interactions (synergistic or antagonistic) can affect plant nutrition, but the effects of deficient or excessive supply of B on mineral uptake and functions are not well established.

There are contrasting results concerning mineral uptake, potentially due to the use of many crop species (Lombin and Bates, 1982), as well as varieties (Mozafar, 1989). Similarly, the use of nutrient solution (Wallace et al., 1977) or diverse soils types (Singh and Sinha, 1976; Agbenin et al., 1991), and the characterization s of different plant parts (Miller and Smith, 1977; Singh and Singh, 1984) at various growth stages (Carpena-Artes and Carpena-Ruiz, 1987) might have contributed to such apparently inconsistent findings. The present review is aimed at critically appraising the available information on the interaction between B and other mineral elements, based on the hypothesis that B (being involved in many physiological and biochemical processes) influences uptake and utilization of other plant nutrients and beneficial elements. We critically discuss the current knowledge about the role of B and its physiological and molecular relationships with other elements, with the aim of laying the groundwork for the identification of relevant interaction networks involved in plant growth and development.

## Interaction of B and macroelements

### Boron interactions with nitrogen

Nitrogen (N) in plants enhances vegetative growth, photosynthetic rates, chlorophyll content, and is an essential mineral nutrient for plant growth and development (Sakuraba, 2022). Nitrogen is a component of proteins, amino acids, nucleotides, and nucleic acids (Koohkan and Maftoun, 2016). Regarding the interaction with B, it has been shown that B is related to N assimilation in plants (Long and Peng, 2023). Furthermore, the interaction of B and N has great importance because of the interference of N in B nutrition, either promoting or reducing the absorption of B in plants (Petridis et al., 2013). In *Vicia faba* L. (faba bean), the interaction between B and N affects the absorption and utilization of N and other nutrients, such as P, K, Ca, and Mg, influencing plant growth in terms of height, leaf area, number of pods, and seed yield (Mahmoud et al., 2006) (**Table 1**). In *Brassica napus* L. (canola), N application in conditions of excess B improve the chlorophyll levels and decrease the severity of B toxicity symptoms (Koohkan and Maftoun, 2016). Recently, the effects of the B x N interaction on winter triticale (*x Triticosecale* Wittmack) productivity have been reported, whereby the application of B increases grain yield and improves the yield components, mainly the number of ears (Bielski et al., 2020). Boron has also been shown to be essential in N_2_ fixation and assimilation regarding nodulation in soybean (*Glycine max*) (**Table 1**) due to the impaired biosynthesis of early nodullin proteins (ENOD2) and malfunction of the oxygen diffusion barrier when B is scarce (Bellaloui et al., 2014). Boron deficiency in the culture medium supporting peas (*Pisum sativum* L.) diminishes symbiotic N_2_ fixation by reducing the number of nodules and interfering with their development, as well as causing disorganization and changes in the cell wall structure (Ahmad et al., 2009).

**Table 1:** Interaction of boron with other minerals in different plant species.

Minerals	Function	Plant species	Deficiency effect of an element	Response	Synergism	Reference
B - Ca	Cell wall biosynthesis, sugar transport, carbohydrate and RNA metabolism Vegetative and productive development of fruits and vegetables	Tomato Peppers	Low B level provoked abnormal changes in the cell wall	Both influence the functions of cell walls, allowing better mobility in the plant At the foliar level, it promotes plant development and increases fruit production in pepper plants	+ +	(Şahin et al., 2015) (Zeist et al., 2018)
B - Al	Decreases toxicity of Al	Pea, Orange	Boron deficiency disrupts Al-induced inhibition of root elongation by accumulation of Al in the transition zone of lateral roots	The entry of Al prevents the solubility of B, inhibiting the production of roots	–	(Li et al., 2018). (Yan et al., 2019) (Yan et al., 2022)
B - N	Fixation of N (formation of nodules) Promotes the absorption and utilization of N and other nutrients	Soybean Tobacco	Boron deficiency causes a drastic decrease in nitrate content and nitrate reductase activity, and increases the content of carbohydrates in leaves from tobacco plants	B improves nitrate levels B in rhizobial N fixation, actinomycete symbiosis and formation of cyanophyte heterocysts in legume crops Additively increases the content of nutrients in plant tissues	+ +	(Bellaloui et al., 2012) (Mahmoud et al., 2006) (Cervilla et al., 2009) (Camacho-Cristóbal and Gonzalez-Fontes, 1999)
B - P	Sugars and cell división	Lettuce Rice Corn and beans	Boron deficiency reduces phosphate absorption capacity, due to reductase activity	Both are involved in the functions of the plasma membrane, influencing cell growth	+	(Petridis et al., 2013) Tariq and Mott, 2007) (Atique-ur-Rehman et al., 2018)
B - K	Buffers and improves cell membrane permeability and protein synthesis Vegetative and reproductive growth	Corn Sunflower Cotton	Boron deficiency decreases permeability to K at the cell membrane	The contribution of both improves the production of grains and leaves With an optimal level of B, the permeability of K in the cell membrane increases Both help to maintain conductive tissues and to exert a regulatory effect on other elements Foliar application of both increases biomass production and cotton yield	+ +	(Rehim et al., 2018) (Samet et al., 2015) (Azeem et al., 2020)
B - Zn	Pollination, seed formation. Intervenes in RNA processes Structural role in the cell wall	Corn Rice Pistachio	Zn deficiency reduces the activity of RNA polymerase Boron deficiency decreases Zn uptake in the plant The lack of B decreases growth and photosynthesis parameters Zinc deficiency reduces stomatal conductance	Between the two, they influence pollination and seed formation B increases growth and chlorophyll content	+ +	(Ziaeyan and Rajaie, 2009) (Tariq and Mott, 2007) (Tavallali, 2017)

Studies performed in canola (*Brassica napus* L.) show negative effects of excess B on plant yield, which could be alleviated with N fertilization (Koohkan and Maftoun, 2016). This result suggests that N might alleviate the growth suppression effects caused by B toxicity, due to the formers positive effects on chlorophyll levels and photosynthesis in canola (*Brassica napus* L.) plants (Koohkan and Maftoun, 2016). Nonetheless, other research groups reporte that the foliar application of B in mango (*Mangifera indica* L.) was effective in improving the nutritional status, since it increases the concentration of N, P and K in the leaf, as well as the levels of chlorophylls and carbohydrates, and the C/N ratio (Shaban et al., 2019).

An important feature of the B x N interaction is the high mobility that both elements possess in soil (Brown and Shelp, 1997; Grohskopf et al., 2020). Several studies suggest that N supply in different concentrations leads to a decrease in B uptake by the plants. Nevertheless, the reported results regarding the effect of N on B deficiency are still controversial and need further investigation (Lou et al., 2003). Thus, at the present time, several studies indicate the presence of many gaps in our knowledge that remain to be elucidated in the B x N interaction.

Likewise, molecular mechanisms, pathways and interactions are still a subject that needs deeper study, as most studies have focused on the improvement and alleviation of B stress at a physiological level (Seeda et al., 2021; Long and Peng, 2023). Nevertheless, as new molecular biology techniques arise together with bioinformatics, it becomes increasingly interesting to try and elucidate the interaction of B with different elements at a molecular level. Thus, it has been reported that B deficiency affects the transcriptional level of genes related to nitrate assimilation (Camacho-Cristóbal et al., 2011; Beato et al., 2014). For example, in root the mRNA concentration of *NRT2* (*High Affinity Nitrate Transporter*) and leaf *NIA* (*Nitrate Reductase*) genes are low in tobacco (*Nicotina tabacum*) plants subjected to severe B deficiency, compared to control samples (Camacho-Cristóbal and Gonzalez-Fontes, 1999; Camacho-Cristóbal and Gonzalez-Fontes, 2007) (**Table 2**). Nonetheless, have in mind that these studies were subjected to a long-term period of B deficiency, therefore, it cannot be ruled out that the changes in gene regulation are an indirect effect due to poor cellular development of the plants. Also, enzymes such as glutamine synthetase and asparagine synthetase, increased transcript levels subjected to B depleted conditions (Beato et al., 2010; Beato et al., 2014), even though these genes could be considered as general responsive genes activated under various abiotic stresses. Another important feature of this interaction at a molecular level is a study carried out by Camacho-Cristóbal and González-Fontes (2007), where they found that short-term B deficiency decreases nitrate content in leaves of tobacco plants, possibly due to a drop in the levels of H^+^-ATPase (PMA2) plasma membrane transcripts. Nonetheless, these findings need to be further investigated.

**Table 2:** Molecular interaction of boron with other minerals in different plant species.

Minerals	Plant	Genes	Response	Reference
B - N	Tobacco	*NtNRT2* (high affinity nitrate transporter) *NtNIA* (nitrate reductase)	Boron can regulate positive or negative nitrate transporters	(Camacho-Cristóbal and Gonzalez-Fontes, 2007)
B - P	Rapeseed	*BnaPT10, BnaPT11*, *BnaPT35* and *BnaPT3* *BnaPHT1* *BnaC3, SPX3*	B could have a role in regulating the expression of P transport genes in roots under low P conditions High supply of B induces the expression of P-starvation *BnaC3*, *SPX3* and the P-transport genes in roots under low P availability.	(Li et al. 2019a; Hua et al., 2017) (Zhao et al., 2020)
B - K	Arabidopsis	*AtAGP13*	B regulate the expression of AGP genes under B deficiency	(Armengaud et al., 2004)
B - Ca	Arabidopsis	*AtCNGC19; AtACA;* *AtCAX*, *AtCNGC19, AtACA* and *AtCAX*	Low B may regulate the expression of *CNGC19*, *ACA* and *CAX3* Ca^2+^ transporter genes and induce an augmented in the cytosolic Ca^2+^, also, it could be attributed to the expression of Ca^2+^ transporters, regulating Ca^2+^ homeostasis in B deficiency.	(Quiles-Pando et al., 2013) (Quiles-Pando et al., 2019)
B - Zn	Arabidopsis Barley	*At1g03770* *HvC2H2*	B could regulate the expression of the *At1g03770* gene that is predicted to encode transcription factors of the zinc finger family, involved in the downstream regulation of genes in response to high B levels. B could regulate the expression of *C2H2* under toxic B conditions	(Kasajima and Fujiwara, 2007) (Pandey et al., 2022)
B - Si	Rice Barley	OsLsi1 (NIP III); HvLsi1/HvNIP2;1	NIP members have been shown to be involved in the uptake of B and Si	(Shao et al., 2018) (Schnurbusch et al., 2010)
B - Al	Citrus	XP_006479398 (*Flavonol synthase/flavanone 3-hydroxylase-like*), NP_197540 (*Flavanone 3 hydroxylase-like*); ADL36732 (*HSF domain class transcription factor*) ATP Binding Cassette (*ABC*)	Gen expression in *Citrus grandis* roots showed that B appears to alleviate Al toxicity Alleviation of B-induced Al toxicity; Regulation of the *ABC* transporter	(Zhou et al., 2015) (Yang et al., 2018)
B - Cd	Rice	*OsHMA2*, *OsHMA3*, and *OsNramp1*, *OsHMA2*, *Nramp1*, and *ABC*	Boron inhibits the expression of these Cd transporters, reducing Cd uptake and transport, decreasing Cd accumulation in aboveground and belowground parts of rice plants.	(Qin et al. 2022) (Riaz et al., 2020; Riaz et al., 2021) (Huang et al., 2021)

### Boron interaction with phosphorus

Phosphorus (P) is an essential macronutrient for plant growth and productivity. This element is a key constituent of macromolecules like nucleic acids, nucleotides and phospholipids of the plasma membrane. Phosphorus is also involved in several biological processes such as protein regulation, photosynthesis, cell division, respiration, and of coenzymes that activate synthesis of amino acids and other compounds (Vance et al., 2003; Paz-Ares et al., 2022) (**Table 1**). The interaction of B x P is not yet clear; nonetheless, borates and phosphates are similar in their action in several physiological and biochemical aspects. For example, both borates and phosphates form physiologically active esters with organic compounds due to their polyhydroxy nature (Atique-ur-Rehman et al., 2018). The uptake and transport of B in plants has been associated with P uptake, because when the concentration of B is low, phosphate uptake decreases, which then recovers when B is supplied (**Table 1**) (Atique-ur-Rehman et al., 2018). A recent study suggests that B supply modulates H^+^-ATPase-mediated plasma membrane nutrient uptake in three species of *Citrus* [*C. sinensis* (L.) Osbeck cv. Valencia, *C. limonia* (L.) Osbeck, and *C. paradisi* Macf. X *Ponsirus trifoliata* (L.) Raf.] (Ferreira et al., 2020). In this sense, B could be related to a reduction in the absorption capacity of phosphate due to the decay of the ATPase activity (Yan et al., 2002).

Furthermore, it has been reported that the synergistic effect between B and P modulates the absorption and distribution of P, as well as the improvement of the photosynthetic rate and growth in *B. napus* plants (Zhao et al., 2020). Another example is the foliar application of B in jojoba plants [*Simmondsia chinensis* (Link) Schneider], where the P level in leaves increases, and where both elements show a significant response in improving plant growth, yield and seed quality under desert conditions (Khattab et al., 2019). In addition, P nutrition mitigates the adverse effects of B toxicity on yield and fruit growth in tomato (*Solanum lycopersicum* L.) plants (Kaya et al., 2009). In this sense, it has been described that P can reduce the harmful effects of B toxicity on plant growth and performance through the reduction of B absorption in tomato (Nejad and Etesami, 2020).

On the other hand, Zhao et al. (2021a) show that the application of B and P displays a synergistic and positive response, by increasing seed yield and phosphorus use efficiency (PUE). Also, sequencing of 16S rRNA genes of bacterial community, reveal that the treatment of B and P increased the diversity of soil bacteria in *B. napus* plants. Furthermore, the effect of *Bacillus pumilus* bacteria on the absorption of B and P after application of both elements improves growth in *B. napus* plants compared with the control (Masood et al., 2019). Moreover, the inoculation of *B. pumilus* improves B levels in *B. napus* plants in B-deficient soils. However, the dicovery of these interactions with biotic and abiotic factors are recent and require further studies to fully understand their effects on different species.

At the molecular level, little it known about the interaction between B x P; however it has been documented that B could play a role in regulating the expression of P transport genes in roots of *B. napus* under low P conditions. Several genes have been identified such as *BnaPT10*, *BnaPT11*, *BnaPT35* and *BnaPT3*, that tend to be simultaneously induced by both P and B deficiencies (Hua et al., 2017; Li et al., 2019a) (**Table 2**). These *BnaPHT1* genes are poorly induced by B and have been detected in B-deficiency transcriptomes in *B. napus* plants (Hua et al., 2017). In a more up-to-date study in this species, the authors suggest that a high supply of B could induce the expression of the P-starvation-induced gene *BnaC3*.*SPX3* (SPX-domain-containing proteins) and the P-transport genes in roots under low P conditions (Zhao et al., 2020). In spite of these findings, the molecular interaccion between B and P transport remains poorly understood.

### Boron interaction with potassium

Potassium (K) is an essential macronutrient for plants, key in several metabolic processes, such as enzyme activation, stomata regulation, balance in the change of anions, and physiological function in plant cell, among others (Fageira, 2001). Nonetheless, little research has been carried out on the interaction between B and K in plants. Studies performed in *B. napus* show a positive correlation between B x K interactions, due to a significant increase in seed oil content and overall oil yield in this crop (Chen et al., 1997). Another study conducted by Liza et al. (2021) reveals that the combined nutrition of B and K results in a significant increase in growth, as well as a 40% rise in yield compared to when the nutrients were provided individually to mung beans (*Vigna radiata* L.). In this context, the authors suggest that whilst K promotes a higher photosynthetic rate, B participates in cell division and cell elongation, so their interaction results in improved plant growth. Similar results were reported by Azeem et al. (2020), where the combined leaf application of B + K fertilization has a positive impact on growth and yield of cotton (*Gossypium hirsutum* L.). This treatment increases biomass production as well as vegetative and reproductive activity under high salinity conditions (**Table 1**). These results could be related to the role of K in osmotic processes, carbohydrate and protein biosynthesis, the closing of stomata, membrane permeability and pH control in plants (Ragel et al. 2019).

Other studies show that B application increases B and K concentrations in rice (*O. sativa* L.), given that B doses increase K permeability in the plasma membrane of the cell (Atique-ur-Rehman et al. 2018). In fact, it is known that B influences the activation of the cell membrane through H^+^-ATPase activity in root cells, as H^+^ pumping drives hyperpolarization of the plasma membrane, thus triggering K uptake to maintain electrochemical balance at the cellular level (Mattos et al., 2017). In tomato plants, Kaya and Ashraf (2015) describe that B toxicity significantly reduces K availability in leaves, as well as that of N and Ca, whilst foliar application of nitric oxide decreases B concentration and augments K, N, and Ca in tomato leaves. In addition, a study focused on the B x K interaction in wheat (*T. aestivum* L.) reports that B toxicity significantly decreases the concentration of K in shoots (El-Shazoly et al., 2019).

The B x K interaction at the molecular level has not been studied exhaustively. A gene expression study using microarrays in *A. thaliana* reports that genes like *AGP13*, *AGP14*, and *AGP22*, are downregulated under B deficiency (Camacho-Cristóbal et al., 2008). Interestingly, *AGP13* (*arabinogalactans, AGP*) transcripts are also downregulated in *A. thaliana* roots during K starvation, even in the absence of fluctuating B levels (Armengaud et al., 2004) (**Table 2**). AGPs are proteins that are distributed differently throughout plant tissues depending on their development, and these may be possible candidates at a cell surface level like signaling across the cell wall, plasma membrane and cytoskeleton (Sardar et al., 2006; Pereira et al., 2014). In this sense, changes in B concentrations may activated a cascade of signals, which may extend through cytoplasm, cell wall, plasma membrane, and cytoskeleton like a continuum, with the possible involvement of such proteins (Goldbach and Wimmer, 2007).

### Boron interaction with calcium

The B x Ca relationship has been observed mainly through the cross-linking of pectin polysaccharides in the plant cell wall (Kobayashi et al., 1999); however, the nature of this interaction is still debated. Several reports suggest a role for Ca in the stabilization of B complexes (B-RG-II), specifically in its ability to bind to carboxyl groups of the polygalacturonic acid regions (Kobayashi et al., 1999; Chormova et al., 2014b; Liu et al., 2019). In fact, a close relationship between B x Ca with respect to cell wall functionality and integrity has been reported, where Ca plays a fundamental role in wall plasticity and elongation, and B is involved in wall metabolism through the maintenance of the Ca-pectin association, influencing the development of the cell wall (Yamauchi et al., 1986). Accordingly, it has been reported that *in vitro* dimerization of pectins such as RG-II are slow, but rise markedly when Ca is applied, as shown in rose (*Rosa* sp) cells in B-free medium (Chormova et al., 2014b). In addition, several studies have presented evidence that Ca^2+^ is a constituent for the formation of borate-RG II complexes, stabilizing the pectic polysaccharides in the cell wall (Kobayashi et al., 1999; Goldbach et al., 2007; Chormova et al., 2014a; Li et al., 2017). Studies performed in pea under salt stress conditions show that the addition of B and Ca positively affect root elongation and plant development (El-Hamdaoui et al., 2003). It has also been observed that the N content in plants originating from seeds is decreased by salt stress and enhanced by B and Ca supply (Bonilla et al., 2004), suggesting an important role of B and Ca in the remobilization of nutrients stored in seed. On the other hand, experiments carried out in pansy (*Viola xwittrockiana* Gams.), petunia (*Petunia xhybrida* hort. Vilm.), and gerbera daisy (*Gerbera jamesonii* Bol. ex Adlam.) show that plants in the absence of Ca or B exhibit discoloration (chlorosis) and upward rolling of leaves, as well as thickening of leaves, distorted meristems, and strap-like leaves, leading ultimately to necrosis (Krug et al., 2009). The authors show that a temporary deficiency of either Ca or B provokes lasting symptoms throughout the whole production cycle, although the symptoms were more similar to those caused by B deficiency than to those that arise due to a lack of Ca. Other studies in radish plants (*Raphanus sativus* L.) report the effect of supplying Ca in ameliorating B toxicity. Indeed, Ca reduces the accumulation of B, and mitigates cellular oxidative damage by enhancing the antioxidant activity of enzymes like superoxide dismutase, catalase, peroxidase, glutathione reductase, and ascorbate peroxidase (Siddiqui et al., 2012). Similar results have been reported by Liu et al. (2019), where Ca reduces B toxicity in trifoliate rootstocks (*Poncirus trifoliate* L) by maintaining the antioxidant enzyme system, and diminishing B concentration in the cell wall and intracellularly. Together, these results suggest that Ca nutrition can be recommended as an agronomic management practice strategy that mitigates B toxicity.

In durum wheat (*Triticum durum* L.), and bread wheat (*Triticum aestivum* L.) genotypes, an assay in plant pots was carried out to evaluate the effects of B application on Ca, showing that high doses of B enhance the concentration and overall quantity of B in leaf cell walls, whereas a fall in cell wall Ca concentration is observed (Turan et al., 2018). This suggests that a negative interaction between Ca x B could decrease B excess in wheat and other related plant species. Nevertheless, further cellular research is required to assess the affinity of Ca and B with respect to crosslinking within the cell wall. In this context, several reports have shown that these two elements, B and Ca, are closely related to each other; consequently, the deficiency or excess of B or Ca can affect the nutritional status of the other, and even of other elements (Krug et al., 2009; González-Fontes et al. 2014; Piñero et al., 2017). Therefore, B and Ca are crucial for plant performance and influence the firmness and quality of seeds and fruits, and consequently it becomes necessary to understand and deepen our knowledge of the interactions of these nutrients at a physiological, biochemical and molecular level.

As stated, B deficiency also affects the expression of genes involved in major physiological processes. However, the signal transduction pathways through which plants are able to sense and transmit B-deprivation signals to the nucleus are unknown. Consequently, a study investigated whether short-term B deficiency in *A. thaliana* roots affects cytosolic Ca levels and signaling. The authors suggest that B deficiency induced an early response of genes such as *CNGC19 Ca^2+^-influx channel*, *ACA*- and *CAX-efflux*, and Ca^2+^ sensor genes, which regulate Ca^2+^ homeostasis (Quiles-Pando et al., 2013). This suggests that gene regulation under B deficiency could enhance the ability to transport Ca^2+^ from the cytosol to plastids, apoplasts, and vacuoles and thus restore cytosolic Ca^2+^ homeostasis (**Table 2**). On the other hand, González-Fontes et al. (2014) reported that at short-term, B deficiency affects cytosolic Ca^2+^ levels, and in roots, upregulates the expression of genes from the MYB protein family involved in Ca^2+^ signaling and represses genes of the bZIP protein family with roles as channels/transporters, sensor relays and responders that act as intermediaries in a transduction pathway triggered by B deficiency, with important consequences in plant development, growth, flower maturation and stress (Zhao et al., 2021b). Another study performed in tobacco plants shows that short-term B deficiency is related with the influx of Ca^2+^ ions and the expression of *WIPK* and *WIZZ*, associated with BY-2 cells and pectin network structure (Koshiba et al., 2009). A more recent study by Quiles-Pando et al. (2019), supports the idea that B deficiency regulates the expression of Ca^2+^ transporter genes such as *CNGC19*, *ACA* and *CAX3*, triggering an increase in the Ca^2+^ concentration in the cytoplasm. This might be attributed to the expression of Ca^2+^ transporters in an attempt to regulate Ca^2+^ homeostasis based on a response due to B deficiency.

## Interaction of B and microelements

### Boron interaction with zinc

The interaction between different nutritional elements is very important in plant nutrition. The B x Zn interaction affects metabolic processes in whole plant either stimulating or inhibiting the uptake of other nutrients hence, effecting the mineral composition In calcareous soils, Hosseini et al. (2007) studied the B x Zn interaction in maize plants (*Zea mays* L.), discovering that Zn significantly increases plant height and dry matter yield, whereas high B levels reduce plant height and dry matter yield, suggesting that the B x Zn interaction was antagonistic on nutrient concentration and synergistic on plant growth. In this case, agronomically it is recommended to add Zn supplements in soils with high B levels, particularly when Zn availability in soil is low. In maize, B and Zn fertilization produce significant changes in some plant nutrients, although these differences were marginal and did not affect plant growth and production (Hosseini et al., 2007). Another study carried out by Tavallali (2017) describes the effects of Zn and B on physiological and biochemical aspects in pistachio plants (*Pistacia vera* L. cv. Badami). This study suggests that high B levels, as well as the lack of B, could reduce growth and photosynthetic parameters (**Table 1**), particularly under low Zn levels. These authors report that Zn deficiency results in a reduction in net photosynthesis (Pn) and stomatal conductance (gs). Nonetheless, the adverse effects of low and high B levels are mitigated by increasing Zn concentration up to 10 mg kg^−1^ soil. In fruit species, B and Zn are important elements for normal fruit growth and development, whose deficiency affects metabolic processes, such as reduced shoot growth, mineral and nutritional alteration, and fruit quality (Marschner, 2012; Özenç and Özenç, 2015; Davarpanah et al., 2016). Foliar application of B and Zn in different doses in European hazelnut (*Corylus avellana*) show that only Zn significantly increases in kernels, and also leads to rises in Ca and Na concentration in leaves (Meriño-Gergichevich et al., 2021). The authors conclude that the foliar application of B and Zn (at 800 and 400 mg L^-1^ respectively) are the most efficient doses for boosting the yield of fruits per plant. On the other hand, in olive cultivars (*Olea europea* L.), foliar application of B and Zn increases phenolic compounds and oil content during the fruit ripening process (Saadati et al., 2013). The oil content increases from 11.7% to 19.4%, highlighting that the applications of B and Zn improve the ratio of unsaturated/saturated fatty acids with respect to the control plants. Moreover, in a soil experiment Quddus et al. (2022) show that different doses of B (0, 1, 2 and 3 kg ha^-1^) and Zn (0, 2, 3 and 4 kg ha^-1^) affect nutrient absorption, yield, and fruit quality of strawberry (*Fragaria x ananassa* Duch.). The doses of 2 kg B ha^-1^ and 3 kg Zn ha^-1^ lead to the highest number and yield of fruits, increase soluble solids and ascorbic acid contents, and B and Zn absorption. These results indicate that the interaction of B x Zn increase the quantity and quality of strawberry fruit. On the other hand, as mentioned above, B and Zn are important microelements for normal plant function (Shrestha et al., 2020; Meriño-Gergichevich et al., 2021; Verma et al., 2021). However, even though their effects have long been investigated in many agronomical and molecular studies, the interaction of B x Zn remains scarce knowledge at genomic and transcriptomic levels.

At a proteomic level, the differential expression of *HKX1* and *MAKR6* genes using the RAPD-PCR method in strawberry plants exposed to combined doses of B x Zn (Kiryakova et al., 2016) was analyzed. The function of the proteins encoded by these genes is mainly related to plant hormones, signal transduction and sugar metabolism (Jing et al., 2020; Novikova et al., 2022), raising interest in such genes whose expression may offer protection during the B x Zn interaction. In another study at the proteomic level, under low and high B conditions in *A. thaliana*, *RING1B* was reportedly induced by high B content in roots, with a locus tag At1g03770 (Kasajima and Fujiwara, 2007). *RING1B* is predicted to encode a Zn finger family transcription factor, and therefore it is possible that this gene regulates the expression of genes that are highly-responsive to B. Besides, *RING1B* has been classified with an important role in the maintenance of shoot stem cell activity (Chen et al., 2010).

Another example is the gene encoding a C2H2 Zn finger transcription factor protein which shows a two-fold upregulation in barley plants under B-toxic conditions (Pandey et al., 2022) (See **Table 2**). This particular gene has been shown to be involved in plant growth and development, stress signal transduction and, more particularly, responses to abiotic stress (Han et al., 2020). Nevertheless, its expression is not upregulated enough to be highly significant. A more up-to-date RNA-seq study shows an important enrichment of three genes of the *C3H* gene family (123068901, 123060371 and 123189473), which belong to a subgroup of the family of Zinc Finger Proteins and are observed under high B conditions in wheat *Triticum dicoccum* shoots (Khan et al., 2023).

### Boron interaction with manganese

Manganese (Mn) is an important element for plant growth and development (Li et al. 2019a). It acts as a cofactor in enzymatic activity, and of the oxygen-evolving complex (OEC) in the photosynthetic machinery in the catalysis of the water-splitting reaction in photosystem II (PSII) (Alejandro et al., 2020). Other functions of Mn are associated with the control of the biosynthesis of the phenolic polymers lignin and suberin, compounds related to the resistance of enzymatic degradation, and avoidance of fungal pathogen invasion in plants (Vidhyasekaran, 2004; Agrios, 2005; Simoglou and Dordas, 2006). In this sense, a work that combined B, Zn and Mn nutrition in coffee (*Coffea arabica* L.) plants showed that all three elements affect the polyphenol concentration, but only Mn increases lignin concentration, reducing the severity of rust on seedlings in nutrient solution (Pérez et al., 2020). In addition, in wheat (*T. aestivum* L.) seedlings, B and Mn applications have significant effects on the reduction of the number of lesions per leaf between booting and milk stages (Simoglou and Dordas, 2006). Furthermore, the combined application of B, Mn and Zn increases in plant growth, shelling ratio and chlorophyll concentration in pea plants due to synergism between the elements (El-Aidy et al., 2021). In another case, antagonistic effects were reported; for example, in tobacco leaves, the increase of B concentrations diminishes the Mn/Fe ratio, due to a rise in the Fe concentration and a fall in Mn levels (Ali et al., 2015).

### Boron interaction with iron

It has been suggested that B promotes the absorption and longdistance transport of Fe in plants (Alvarez-Tinaut, 1980). In tomato growing hydroponically, B levels influence Fe absorption and translocation paralleling the dry matter production. Fe absorption varied with B supply in the same way and in a similar pattern to growth under the same B levels (Alvarez-Tinaut, 1980). This points to an indirect influence of B on Fe absorption, through increasing growth and hence Fe (and other nutrients too) demands. Another interaction between B and Fe has been reported in the reallocation of apoplastic Fe in root, an essential Fe storage pool in plants. It is known that B can affect the dimerization of pectin rhamnogalacturonan-II (O’Neill et al., 2004). Peng et al. (2021) reported that a decreased the abundance of the rhamnogalacturonan-II dimer compromised the reallocation of Fe from roots to shoots and severely impaired root growth. This information suggest that B can regulate the chelation of Fe by the cell wall, by its role in the cell wall biosynthesis and thus apoplastic Fe reallocation.

## Beneficial elements and toxic elements

### Boron interaction with silicon

Silicon (Si) is a beneficial element for plants, which has been demonstrated by several studies in many species and environmental conditions (Rizwan et al., 2015; Debona et al., 2017; Etesami and Jeong, 2018; Pavlovic et al., 2021; Song et al., 2021). In barley (*Hordeum vulgare* L.), Akcay and Erkan (2016) described that the combined application of B and Si increased the transcription levels of *BOR2* transporter efflux gene, involved in the B detoxification in the apoplast. Interestingly, the same authors described higher expression levels in the shoot in comparison to the root which could explain the preventive role of the B accumulation and the increased tolerance to high B (Miwa and Fujiwara, 2010). Accordingly, Akcay and Erkan (2016) showed that exist a certain degree of competence in the B transport system that favors Si uptake, being also the mechanism proposed in oilseed rape grown under B excess (Liang and Shen, 1994). In fact, B can be transported through the multifunctional *HvNIP2;1* transporter in barley and rice plants (Schnurbusch et al., 2010; Mitani-Ueno et al., 2011) (**Table 2**). *HvNIP2;1* transporter is the homolog of *OsLsi* , an influx Si transporter, suggesting that both elements use the same transporter system in plants. In addition, a genome-wide association mapping supports the idea that *HvLsi6* is required for efficient B transport in barley (Jia et al., 2021).

### Boron and aluminum in plants

Aluminum (Al) is also a non-functional element in plants. The interaction between B and Al has been proposed to be beneficial, with B promoting the efflux of H^+^ thus regulating H^+^-ATPase activity in the plasma membrane, and reducing the demethylation of pectin to weaken Al binding to carboxyl groups; nevertheless, the processes and mechanism involved in alleviating Al toxicity are still not clear (Li et al., 2017; Li et al., 2018; Yan et al., 2021). Aluminum binds to the cell wall and induces changes in the content, proportion, and structure of cell wall components, particularly in pectin and hemicellulose fractions (Zhou et al., 2015; Xu et al., 2022; Yan et al., 2022). Furthermore, Al has been found to alter the extensibility, rigidity, and porosity of the cell wall (Illés et al., 2006; Zhou et al., 2015; Yan et al., 2022). In *Poncirus trifoliata* (trifoliate orange), it was reported that B application decreases the levels of hydrogen peroxide (H_2_O_2_), malondialdehyde (MDA), and lignin contents in roots of Al-treated plants (Yan et al., 2022). These results suggest that B could be involved in a mechanism that prevents the inhibitory effects of Al on root growth.

Among the various components of the cell wall, lignin is important as it is associated with mechanical properties and is a vital indicator used to assess Al tolerance in plants (Wang and Kao, 2006; Smith et al., 2011). In tree species, it was observed that B-deficiency induces the upregulation of lignin monomer biosynthesis, via the modification in the expression of several transcription factors, including *MYBs*, *WRKYs* and *NACs* in Norway spruce (*Picea abies* L.). On the other hand, in poplar (*Populus tremula* L.), *PtrMYBs* are upregulated under B-deficiency, transcription factors that are orthologues of *AtMYB58* and *AtMYB63*, which are regulators of lignin synthesis (Su et al., 2019). Additionally, plants under B-starvation display changes in the phenylalanine metabolic pathway, which promotes lignin accumulation, suggesting that B is related to lignin content and its metabolic pathway in the cell wall (Wu et al., 2017). It could also be suggested that the effect of B in alleviating Al toxicity is mainly due to the formation of RGII-B complexes, which help to stabilize the cell wall (Li et al., 2017). In this regard, B increases the content of RG-II (KDO, 2-keto-3-deoxyoctonic acid) to create more borate-RGII complexes, and in turn reduces the methyl esterification of pectin, thus forming more negative charges to immobilize Al^3+^ in cell wall pectin. In fact, Al binds to the negatively-charged carboxyl groups of pectins, and both Al-induced ROS and free Al^3+^ can disrupt the cell wall, producing modifications that could in turn reduce elasticity, due to the cleavage of polysaccharides or methyl esterification (Yang et al., 2010; Ranjan et al., 2021). However, when B is applied, it binds to pectin hence reducing the entry of Al to the cell and minimizing the toxic effects of Al (Riaz et al., 2019). In many plant species, the plasma membrane H^+^-ATPase has been studied and Al toxicity can affect both its expression and post-translational activity (Zhang et al., 2017). In this regard, the work of Yan et al. (2021) shows that B could alleviate the Al-induced inhibition in the activity of the H^+^-ATPase by promoting the activity of the H^+^-ATPase and thus H^+^ efflux, therefore weakening the acidic intracellular environment produced by Al. In this case, B also lowers the synthesis of pectin and the activity of pectin methylesterase. This latter point is important to highlight as the degree of methylation of pectin helps determine the amount of carboxyl groups that can bind to Al^3+^ and its sensitivity in different plant species (Horst et al., 2010). It has also been proposed that B promotes alkalization of the root surface of peas. This is regulated by *Polar Auxin Transport* (*PAT*), leading to the downstream regulation of the H^+^-ATPase, consequently alleviating any toxic effect produced by Al (Li et al., 2018).

Additional effects of B on plants in response to Al stress have been described. Working with seedlings of trifoliate orange, Riaz et al. (2018) report changes at a physiological and molecular level, observing differences in root length and improved antioxidant activity based on the alleviation that B produces in interaction with Al. As described in this work, this improvement is thought to be produced because the supply of B reduces the uptake of Al in roots and leaves in response to oxidative damage. According to Yan et al. (2019), in the same species, B can also reduce Al-driven ascorbate synthesis, by downregulating the metabolites involved in the L-galactose pathway. This is believed to be achieved as B eases the effects of Al by decreasing the redox status and activities in the ascorbate-glutathione cycle, via its enzymes ascorbate peroxidase, dehydroascorbate reductase, glutathione reductase, and glutathione peroxidase.

The molecular mechanisms that underlie the B-induced alleviation of Al-toxicity in plants are poorly understood. Studies investigating the gene expression patterns in Sour pummelo (*Citrus grandis*) roots that respond to B x Al interactions show that B appears to alleviate Al toxicity by improving the overall ability to remove ROS and aldehydes, increasing expression levels of lipid-related genes and upregulating cellular transport-related gene expression (Zhou et al., 2015). Another study of the B x Al interaction performed in *C. grandis* supports the alleviation of B-induced Al toxicity by finding that it could be attributed to cell wall remodeling by reducing lignin synthesis (via the sugar ATP Binding Cassette (ABC) transporter ATPase) and increasing the modification of cell wall. Greater abundance of stress response proteins, greater cellular regulation and signal transduction (calreticulin-1) confer a possible mechanism for the alleviation of Al toxicity induced by B (Yang et al., 2018). More recent studies report that B increases the expression of genes (*OsSTAR1* and *OsSTAR2*) that are responsible for reducing the Al content in cell walls in rice (**Table 2**). Furthermore, it significantly increases the expression of *OsALS1*, thus facilitating the transfer of Al from the cytoplasm to the vacuole (Zhu et al., 2019). A transcriptomic study also reports that B could lessen Al toxicity by inducing the expression of several genes, including *PtALMT4* and *PtALMT9*, *PtALS1* and *PtALS3*, and *PtSTAR1*, which is responsible for reducing Al deposition of the cell wall in trifoliate orange plants subjected to Al toxicity (Yan et al., 2022). Based on these reports, B could actually be responsible for regulating several genes and pathways sensitive to Al, reducing the distribution of Al in the subcellular components after its addition.

Therefore, there are several interesting aspects of plant response mechanisms to Al toxicity; nevertheless, more research is needed to identify molecular players associated with B and Al in different species. Indeed, important questions regarding B x Al, such as deacidification, signaling pathways and the global up- and down-regulation of genes through transcriptomics need to be further investigated.

### Boron and cadmium in plants

Cadmium (Cd) is a highly toxic heavy metal for plants (Al-Khayri et al., 2023). At toxic levels, Cd alters the growth, development, yield and quality of plants. The symptoms of Cd toxicity are easily identifiable as chlorosis that occurs due to blocked Fe and Zn uptake, and stunted growth. Cadmium toxicity leads to a greater production of ROS and to a decrease in the chlorophyll content and photosynthetic activity (Nazar et al., 2012; Haider et al., 2021). Regarding the B x Cd interaction, the effect has been reported to occur in the structural and functional integrity of the cell wall and membranes (Nishizono et al., 1987; Riaz et al. 2020). Studies done with B have pointed out that the presence of B in fertilizers could actually mitigate the toxic effects of Cd on crops by enhancing Cd chelation onto plant cell walls (Qin et al., 2020; Wu et al., 2020b; Long and Peng, 2023). According to Chen et al. (2019), B affected favorably the antioxidant machinery in rice, increasing the activities of superoxide dismutase, peroxidase and catalase, mitigating the detrimental effects of Cd-stress. Most of the studies of the B x Cd interaction have focused on rice and oilseed rape, such that diversifying our studies would give more insights about the mitigating effects that B has on Cd in more diverse species.

Several authors have reported that B can mitigate Cd toxicity in plants given that B affects cell wall structures and some components that allow blocking the entry of Cd into the cytosol (Wu et al., 2020a; Wu et al., 2020b; Riaz et al., 2021). Several studies showed that B could significantly reduce the Cd accumulation in roots rice through the downregulating of *Nramp1*, *Nramp5*, *HMA2*, and *HMA4* expression of Cd-induced transporters, promoting the adsorption of Cd in cell wall of roots, and activating the antioxidant enzyme system (Chen et al., 2019; Riaz et al., 2020; Riaz et al., 2021). The repressed expression of these Cd transporter genes by both B and Cd are linked to the reduction of Cd uptake and transportation, diminished Cd accumulation in both aboveground and belowground level in rice plants (Huang et al., 2021). It is thought that B decreases the expression of some Cd-induced transporter genes such as *HMA2*, *NRAMP1* and some *ABC* genes; hence, relieving Cd toxicity and its accumulation in rice seedlings by restraining its uptake and translocation from root to shoot, improves the tolerance and chelation ability that rice can have toward Cd (**Table 2**). In wheat, the expression of Cd genes (*TCONS1113*, *TRIAE1060*, *TRIAE5370* and *TRIAE5770*) in the presence of B was also boosted (Qin et al., 2022), proposed that the application of B could inhibit significantly Cd uptake and translocation through the regulation of Cd transporter genes either at the seedling or elongation phase.

**Figure 1:**
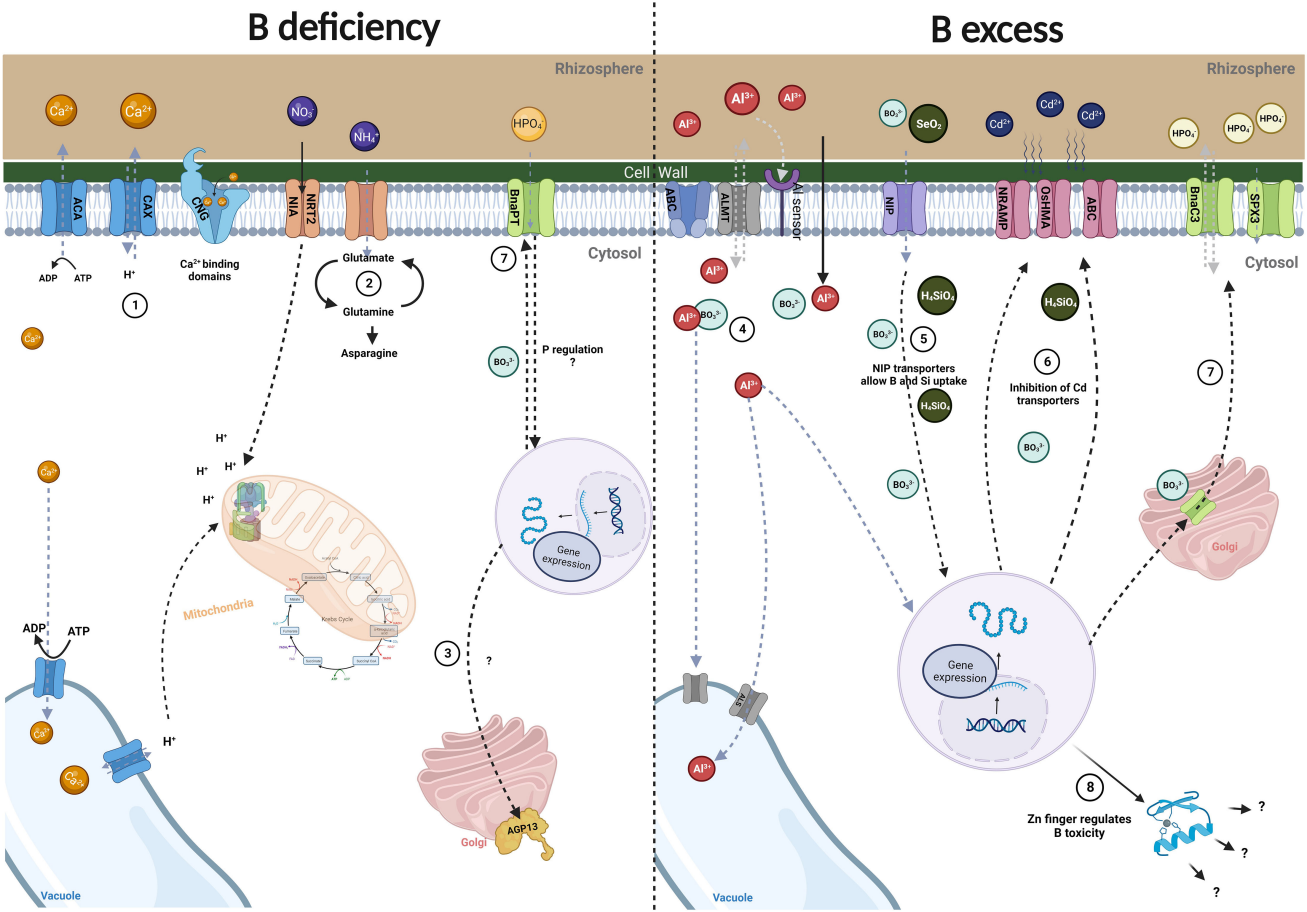
Molecular interaction of B with other elements. (1). Boron interaction with Ca^2+^. Boron deficiency has been associated with changes in the expression of Ca^2+^ genes (*ACA*, *CAX*, *CNCG*) that are activated to restore Ca^2+^ homeostasis within the cytosol. (2) Boron interaction with N. Under B deficiency, nitrate transporters are downregulated affecting H^+^-ATPase transcripts and leading to ammonium accumulation along with elevated glutamine and asparagine production. (3) Boron interaction with K. *AGP* transcripts have been studied in the B x K relationship. Under B deficiency, these proteins are downregulated leading to changes in the membrane-cytoskeleton continuum in which an unknown cascade of signals is thought to be activated. (4) Boron interaction with Al. B has been studied as an alleviator of Al toxicity. Through different mechanisms and regulation of transport-related genes, B induces protein expression to reduce the deposition of Al in the cell wall and diminish its toxicity by importing it into vacuoles. (5) Boron interaction with Si. Si and B interact using the same NIP transporters, possibly allowing for B detoxification when found in high levels. (6) Boron interaction with Cd. Interestingly, B and Si when combined display an inhibitory activity over Cd transporters, accounting for the elimination of Cd toxicity. (7) Boron interaction with P. Under low B conditions, changes in *BnaPT* transporter expression regulates P content. Otherwise, under B toxicity and low P conditions, the transporters *BnaC3* and *SPX3* are upregulated to balance P content. (8) Boron interaction with Zn. Zinc finger proteins are upregulated in response to B toxicity. It is believed that these proteins regulate B content by stress signal transduction pathways to improve plant growth and development. These observations have been studied in different plant species and are not necessarily equivalent in all species. Created with Biorender.

The molecular interactions that B exerts with different elements still need to be elucidated, particularly as these interactions may vary between species. **Figure 1** shows a general scheme of the molecular interactions of B with other elements, in both deficient and excess conditions.

## Conclusions and future perspectives

Boron can be present at insufficient or excessive levels in the soil. The means by which plants cope with such differences requires a study of intra- and inter-species genetic variability, together with new discoveries about the mechanisms of tolerance to B toxicity, that could facilitate the breeding of new varieties with satisfactory yields in soils with high levels of B. Nevertheless, several lines of evidence indicate that extreme deficient or toxic levels of B may be responsible for secondary effects related with impaired plant growth, insufficient nutrient uptake and altered nutrient homeostasis due to interactions with B which can be direct or indirect with other plant nutrients. Furthermore, the interactions of B with other plant nutrients are highly complex and their effects can be antagonistic or synergistic, depending on plant species/varieties and the environment. Environmental factors may provoke B deficiency even in the presence of higher quantities of B in the soil. Moreover, B addition through fertilization in some cases, could enhance crop productivity by alleviating metabolic alterations displayed by toxic levels of Al and heavy metals like Cd, reducing overall yield losses. Since application of B in fertilizers is highly cost-effective, its use in fertilization programs should be properly evaluated alongside a determination that could define whether application of B is more beneficial when added to the soil or to the leaves. In all, B plays important roles in the nutrient interactions within plants; however, important basic questions related with B being directly or indirectly involved when interacting with certain nutrients deserve further research efforts.

## Author contributions

PV-M: Writing – original draft. FA: Writing – review & editing. MR-D: Writing – review & editing. PC-F: Formal Analysis, Writing – review & editing. BS-C: Writing – review & editing. AN-N: Writing – review & editing. CI-B: Conceptualization, Funding acquisition, Project administration, Resources, Supervision, Writing – original draft, Writing – review & editing.
