# Erratum: Role of boron and its interaction with other elements in plants

**DOI:** 10.3389/fpls.2024.1425825

**Published:** 2024-06-05

**Authors:** Frontiers Production Office

**Affiliations:** Frontiers Media SA, Lausanne, Switzerland

**Keywords:** boron, interaction, mineral elements, low pH, protein transport, oxidative stress

**Text Correction**

Due to a production error, some text remained from an earlier version of the manuscript.

A correction has been made to the section **Abstract**, Paragraph Number One. The sentence:

“In this review, we discuss the mechanisms of B uptake, absorption, and accumulation and its interactions with other elements, and how it contributes to the adaptation of plants to different environmental conditions.”

has been replaced with the correct:

“In this review, we discuss the mechanisms of B uptake, translocation, and accumulation and its interactions with other elements, and how it contributes to the adaptation of plants to different environmental conditions.”

A correction has been made to the section **Introduction**, Paragraph Number One. The sentence:

“Boron is considered the most mobile, and often one of the most deficient, microelements in soils…”

has been replaced with the correct:

“Boron is considered as the most mobile, and often one of the most deficient, microelements in soils…”

A correction has been made to the section **Introduction**, Paragraph Number Two. The sentence:

“Boron is a microelement and its concentration in dried leaf tissue varies from 10 to 75 mg kg-1…”

has been replaced with the correct:

“Boron is a microelement and its concentration in dried leaf tissue varies depending on species and genotypes…”

A correction has been made to the section **Interaction of B and macroelements**, subsection **Boron interaction with potassium**, Paragraph Number One. The sentence:

“Nonetheless, little research has been carried out on the interaction between B x K in plants.”

has been replaced with the correct:

“Nonetheless, little research has been carried out on the interaction between B and K in plants.”

A correction has been made to the section **Interaction of B and macroelements**, subsection **Boron interaction with potassium**, Paragraph Number Two. The sentences:

“Furthermore, the effect of salicylic acid on the amelioration of B toxicity was evaluated (Nawaz et al., 2020), indicating that excess B significantly decreases K content in shoots. Nevertheless, these authors also found an increase in K concentration in roots. It is worth mentioning that B excess can coexist with other abiotic stresses, e.g. salt and drought, conditions found mainly in arid and semiarid conditions.”

have been removed.

A correction has been made to the section **Interaction of B and macroelements**, subsection **Boron interaction with calcium**, Paragraph Number Three. The sentence:

“These results agree with those proposed by Gonzaílez-Fontes et al. (2014) where shortterm

B deficiency affects cytosolic Ca2+ levels, and in roots, upregulates the expression of genes from the MYB protein family involved in Ca2+ signaling and represses genes of the bZIP protein family with roles as channels/transporters, sensor relays and responders that act as intermediaries in a transduction pathway triggered by B deficiency, with important consequences in plant development, growth, flower maturation and stress.”

has been replaced with the correct:

““On the other hand, Gonzáles-Fontes et al. (2014) reported that at short-term, B deficiency affects cytosolic Ca2+ levels, and in roots, upregulates the expression of genes from the MYB protein family involved in Ca2+ signaling and represses genes of the bZIP protein family with roles as channels/transporters, sensor relays and responders that act as intermediaries in a transduction pathway triggered by B deficiency, with important consequences in plant development, growth, flower maturation and stress.”

A correction has been made to the section **Interaction of B and microelements**, immediately following subsection **Boron interaction with manganese**, creating subsection **Boron interaction with iron.** The following lines:

“**Boron interaction with iron**

It has been suggested that B promotes the absorption and long-distance transport of Fe in plants (Alvarez-Tinaut, 1980). In tomato growing hydroponically, B levels influence Fe absorption and translocation paralleling the dry matter production. Fe absorption varied with B supply in the same way and in a similar pattern to growth under the same B levels (Alvarez-Tinaut, 1980). This points to an indirect influence of B on Fe absorption, through increasing growth and hence Fe (and other nutrients too) demands. Another interaction between B and Fe has been reported in the reallocation of apoplastic Fe in root, an essential Fe storage pool in plants. It is known that B can affect the dimerization of pectin rhamnogalacturonan-II (O’Neill et al., 2004). Peng et al. (2021) reported that a decreased the abundance of the rhamnogalacturonan-II dimer compromised the reallocation of Fe from roots to shoots and severely impaired root growth. This information suggest that B can regulate the chelation of Fe by the cell wall, by its role in the cell wall biosynthesis and thus apoplastic Fe reallocation.”

were added to this new subsection.

A correction has been made to the section **Non-functional elements**, which has been renamed **Beneficial elements and toxic elements.**

A correction has been made to the section **Beneficial elements and toxic elements** (previously **Non-functional elements**) subsection **Boron interaction with silicon**, Paragraph Number One. The sentence:

“In fact, B can be transported through the multifunctional HvNIP2;1 transporter (homolog of

OsLsi1) in barley and rice plants (Schnurbusch et al., 2010; Mitani-Ueno et al., 2011) (**Table 2**). Genome-wide association mapping supports the idea that HvLsi6 is required for efficient B transport in barley (Jia et al., 2021).”

has been replaced with the correct:

“In fact, B can be transported through the multifunctional HvNIP2;1 transporter in barley and rice plants (Schnurbusch et al., 2010; Mitani-Ueno et al., 2011) (**Table 2**). HvNIP2;1 transporter is the homolog of OsLsi, an influx Si transporter, suggesting that both elements use the same transporter system in plants. In addition, a genome-wide association mapping supports the idea that HvLsi6 is required for efficient B transport in barley (Jia et al., 2021).”

**Error in Table**

Due to a production error, there was a mistake in **Table 2**, Row B-N, Column Response, as published. The sentence:

“The content of B activates or deactivates nitrate transporters”

has been replaced with the correct:

“Boron can regulate positive or negative nitrate transporters”

The corrected **Table 2** appears below.

**Table 2:** Molecular interaction of boron with other minerals in different plant species.

Minerals	Plant	Genes	Response	Reference
B - N	Tobacco	*NtNRT2* (high affinity nitrate transporter) *NtNIA* (nitrate reductase)	Boron can regulate positive or negative nitrate transporters	(Camacho-Cristóbal and González, 2007)
B - P	Rapeseed	*BnaPT10*, *BnaPT11*, *BnaPT35* and *BnaPT3* *BnaPHT1* *BnaC3*, *SPX3*	B could have a role in regulating the expression of P transport genes in roots under low P conditions High supply of B induces the expression of P-starvation *BnaC3*, *SPX3* and the P-transport genes in roots under low P availability.	(Li et al., 2019a; Hua et al., 2017 (Zhao et al., 2020)
B - K	Arabidopsis	*AtAGP13*	B regulate the expression of AGP genes under B deficiency	(Armengaud et al., 2004)
B - Ca	Arabidopsis	*AtCNGC19*; *AtACA*; *AtCAX* *AtCNGC19*, *AtACA* and *AtCAX*	Low B may regulate the expression of *CNGC19*, *ACA* and *CAX3* Ca^2+^ transporter genes and induce an augmented in the cytosolic Ca^2+^, also, it could be attributed to the expression of Ca^2+^ transporters, regulating Ca^2+^ homeostasis in B deficiency.	(Quiles-Pando et al., 2013) (Quiles-Pando et al., 2019)
B - Zn	Arabidopsis Barley	*At1g03770* *HvC2H2*	B could regulate the expression of the *At1g03770* gene that is predicted to encode transcription factors of the zinc finger family, involved in the downstream regulation of genes in response to high B levels. B could regulate the expression of *C2H2* under toxic B conditions	(Kasijama and Fujiwara, 2007) (Pandey and Khan, 2022)
B - Si	Rice Barley	*OsLsi1* (*NIP III*); *HvLsi1*/*HvNIP2;1*	NIP members have been shown to be involved in the uptake of B and Si	(Shao et al., 2018) (Schnurbusch et al., 2010)
B - Al	Citrus	XP_006479398 (*Flavonol synthase/flavanone 3-hydroxylase-like*), NP_197540 (*Flavanone 3 hydroxylase-like*); ADL36732 (*HSF domain class transcription factor*) ATP Binding Cassette (*ABC*)	Gene expression in *Citrus grandis* roots showed that B appears to alleviate Al toxicity Alleviation of B-induced Al toxicity; Regulation of the *ABC* transporter	(Zhou et al., 2015) (Yang et al., 2018)
B - Cd	Rice	*OsHMA2*, *OsHMA3*, and *OsNramp1*, *OsHMA2*, *Nramp1*, and *ABC*	Boron inhibits the expression of these Cd transporters, reducing Cd uptake and transport, decreasing Cd accumulation in aboveground and belowground parts of rice plants.	(Chen et al., 2020) (Riaz et al., 2020; 2021) (Huang et al., 2021)

The publisher apologizes for this mistake. The original version of this article has been updated.
